# Retraction: Exploring the Thoracolumbar Interfascial Plane (TLIP) Block as a Novel Approach for Improved Pain Management After Spine Surgery: A Comparative Review

**DOI:** 10.7759/cureus.r148

**Published:** 2024-09-27

**Authors:** Sweta J Gajapure, Vivek Chakole

**Affiliations:** 1 Anesthesiology, Jawaharlal Nehru Medical College, Datta Meghe Institute of Higher Education and Research, Wardha, IND

This article has been retracted by the Editors-in-Chief due to the citation of a work retracted due to data fabrication. The following article was previously retracted due to data fabrication: 

Ueshima H, Otake H: Clinical experiences of ultrasound-guided lateral thoracolumbar interfascial plane (TLIP) block [RETRACTED]. J Clin Anesth. 2017, 39:145. 10.1016/j.jclinane.2017.04.006

Cureus permits the citation of retracted work provided that the scientific content contained therein is still determined to be credible and accurate. In this case, this threshold has not been met and the citation of the above article should not have been permitted. In addition, the authors misrepresented another cited work (reference 14). The authors were provided with the opportunity to provide an amended section of their article to be used for a correction, but have neglected to so. As a result, the journal has no choice but to retract the article in order to ensure the accuracy of the scientific record.

